# Tables of the Natural System of Medical Science, and of the First Principles of the Medical Art

**Published:** 1808-07

**Authors:** Alexander Walker

**Affiliations:** Lecturer on Physiology at Edinburgh


### TABLES OF THE NATURAL SYSTEM OF MEDICAL SCIENCE, AND OF THE FIRST PRINCIPLES OF THE MEDICAL ART.

PROPOSED BY ALEXANDER WALKER, ESQ.

LECTURER ON PHYSIOLOGY AT EDINBURGH.

THE following Sketch of a Natural System of Medical Science is founded on the strict basis of physiology, and is, I trust, calculated to place, in a new and more interesting point of view, the beautiful relations which subsist between the sciences of anatomy and physiology, and the art of medicine, as well as to lay the foundation of a more rational system of medical practice.—I do not, however, make this statement without a feeling of the utmost deference to the labours of the celebrated Cullen and the more illustrious Brown, the immortal authors of existing systems. Nothing less than a conviction of the truth and simplicity of the system I am about to propose could have induced me to presume to exhibit it.

In it, as I have subsequently observed, it will be seen that all the parts of Medical Science correctly correspond and beautifully flow from each other: while Anatomy is the basis of Physiology, Physiology is the basis of Medicine; the classification of one is applicable to the rest, and all of them at once involve Mineral, Vegetable, and Animal Nature.

A detailed account of this system will shortly be published.

The present Sketch is addressed as a Thesis to my Physiological Class, and to those Medical Gentlemen who may choose to investigate the subject.

To the gentleman who, on the 1st. day of January, 1809, transmits to me a Thesis, exhibiting the greatest number of diseases and of articles of the materia medica, arranged under their respective genera in the Natural System, a Case of Surgical Instruments of Fifteen Guineas in value, with an honorary inscription, will be presented; to the gentleman who exhibits the second greatest number, a Case of Ten Guineas in value will be presented; and to him who exhibits the third greatest numder, one of Five Guineas in value will be presented.—These premia will be adjudged by a committee of medical gentlemen.—To prevent the possibility of partial decisions, mottos only, and not the

 names names of their authors, must be inscribed upon the Thesis, while these must be accompanied by sealed papers, having the corresponding mottos on their outside, and enclosing the names of the authors. These letters shall not be opened unless they bear a motto, corresponding to the motto of a Thesis, to the author of which a premium is decreed.

As the subsequent Tables, therefore, are intended as Theses, neither individul diseases, nor individual articles of the materia medica, are inscribed under them. In order, however, to render their meaning perfectly obvious, it may be necessary to give one or two examples of this mode of classification: Thus, with regard to diseases, Fracture is, according to it, a disease of the Mechanical Functions, Syphilis of the Vital Functions, and Delirium of the intellectual Functions: Further, under this class of disease, Fatuitas is a disease of Diminished Mental Operation, Mania of Depraved Mental Operation, and Vigilia of Increased Mental Operation; and, with regard to Medicines, Caustic affects the Mechanical, Mercury the Vital, Opium the Intellectual Functions, &c.

*St. Anne's Park, Edinburgh,*

*March* 1, 1808.

---

### Division I.

### *Present State of Medical Science.*

I cannot better explain my opinion of the present state of Medicine than by using, respecting it, the language of Lord Bacon, which is, at this moment, as applicable to it, as it was when first written. "We see," says he, "the weakness and credulity of men is such, as they will often prefer a mountebank or witch before a learned physician; and, therefore, the poets were clear sighted in discerning this extreme folly when they made Æsculapius and Circe, brother and sister, both children of the sun.—For in all times, in the opinion of the multitude, witches, and old women, and imposters, have had a competition with physicians. And what followeth? Even this; that physicians say to themselves, as Solomon expresseth it upon a higher occasion; If it befall to me as befalleth to the fools, why should I labour to be more wise? And, therefore, I cannot much blame physicians, that they use commonly to intend some other art or practice which they study more than their own profession. For, you shall have of them, antiquaries, poets, humanists, statesmen, merchants, divines, and and in every of these, better seen than in their own profession; and, no doubt, upon this ground, that they find that mediocrity and excellency in their art maketh no difference in profit or reputation toward their fortune; for the weakness of patients, and sweetness of life, and nature of hope, maketh men depend upon physicians with all their defects."

But Lord Bacon has assigned an amply sufficient reason for these circumstances. In another place he says, "Medicine is a science which hath been, as we have said, more professed than laboured, and yet more laboured than advanced; the labour having been, in my judgement, rather in circle than in progression. For I find much iteration, but small addition." Now it is, in reality, this profession without labour, this labour without advancement, so well expressed by Lord Bacon, this iteration without addition, which is the real and sufficient cause (overlooked by him) of the poets so properly making Æsculapius and Circe, brother and sister, and of the world uniformly placing witches, old women, and impostors, in competition with physicians. And again his Lordship, with equal justice, says, "In the inquiry of diseases they do abandon the cures of many, some as in their nature incurable, and others as past the period of cure; so that Sylla and the Triumvirs never proscribed so many men to die, as they do by their ignorant edicts, whereof numbers do escape with less difficulty than they did in the Roman proscriptions. Therefore I will not doubt to note as a deficience, that they inquire not the perfect cures of many diseases, or extremities of diseases; but pronouncing them incurable do enact a law of neglect, and exempt ignorance from discredit."

It, therefore, becomes proper to ascend to the cause of the little success which has hitherto attended medical investigation; and to attempt, by the removal of that cause, to accelerate its progress; and, by increasing the success, to increase the respectability of physicians.

### Division II.

### *Causes of the present State of Medicine.*

The grand cause which has hitherto retarded the progress of medicine, has been the total want of a general principle, and this want has arisen from the very low state of physiological theory.

It has ever appeared to me that a sufficient distinction has not been made between diseases as affecting various functions, functions, which, to the most superficial observer, seem to be very different in their nature.—It would appear at first sight, that, instead of almost uniformly applying mental remedies to mental diseases, the physician not only does not distinguish what stages and parts of the same disease are mechanical, what vital, and what intellectual, which must uniformly be the case where all the functions are involved, but that he does not clearly perceive such a distinction at all to subsist in any form or combination of disease.

The physician has also failed to attend to a correct arrangement of the various articles of the materia medica, which ought not to be considered merely as stimulant, sedative, &c. but as, in reality, operating upon different functions.

Besides these, there appear to be other subordinate objects of inquiry, which he has hitherto, to a certain extent, overlooked; namely, that medicines seem to be objectionable from the injury which they do to those functions that are the vehicles of their operations, the uncertainty of their effects as influenced by association, the uncertainty of their continuance as dependant on the same and other causes, the difficulty of removing their bad effects, and the injury they do to the intellectual functions, in making man entirely the creature of direct impression, instead of reflection.

These seem to me points eminently worthy of consideration, and the best mode of obviating the objections they involve, is a greater attention to what I should term mental remedies.

### Division III.

### *Mode of obviating the present State of Medicine.*

But in order perfectly to remedy these great defects, the cultivation of physiology is primarily necessary. As far as the accumulation of facts go, it has, however, already been cultivated, and that most assiduously. But before these accumulated facts can afford a principle of medicine, they must afford a theory of physiology; and, before they can afford this, they must be arranged according to their relations to each other.

I may here observe, that the sciences alone afford theories; the arts admit merely of principles upon which these reasonings or theories are applied to the wants of man. Thus in every step of their progress, the arts must implicitly depend upon the sciences.

Now

Now medicine is purely an art, and admits of a principle derived from physiology, by which the theories of that science may be applied to the cure of diseases.

As, however, no general theory of physiology exists, it becomes necessary to inquire what systematic arrangement can be given to its facts as strictly founded on those of anatomy, in order to obtain one. And I know none so unexceptionable as that which I am about to deliver, nor any general theory so rational as the one which results from that arragement. I shall first, therefore, exhibit these arrangements, and then try their applicability to the medical art.

### Division IV.

### *Natural Arrangement of Medicine.*

In proceeding to a natural arrangement of *anatomy* and *physiology*, it appears to me an unquestionable point, that it ought to indicate, at a single glance, the dependence of one function upon another. It is upon this principle that I have constructed the natural arrangement which I am about to detail. And a single remark on the arrangements adopted by the most celebrated modern physiologists will, at once, shew the originality of the plan which I propose, and point out the errors of arrangement which I deprecate.

Blumenbach, Chausier, Dumas, and Bichat treat of circulation before absorption, on which it depends; and even before mastication, deglutition and digestion, on which, in general, even absorption chiefly depends.

If the arrangement of Chausier in particular, who, in this respect, is as accurate as any of them, were to be considered as indicating the *relation* and *dependance* of the functions, so absurd is it, that absorption, instead of the cause, would be the result of nutrition, generation the result of absorption, and digestion the result of generation.

ANATOMY, I divide into three parts; namely, that which considers the *Mechanical* or *Loco-motive Organs*, that which considers the *Vital Organs*, and that which considers the *Intellectual Organs.*—Under the Mechanical or Loco-motive Organs, I class, first, the *Bones*, which support the rest of the Animal Structure; second, the *Ligaments*, which unite them; and third, the Muscles, which move them.—Under the Vital Organs, I class first, the Organs of Digestion, the Absorbent Surfaces, and the Vessels which absorb from these surfaces; second, the

*Heart*,

*Heart*, *Lungs*, and *Blood-vessels*, which derive their contents (the blood) from the absorbed Lymph; and third, the *Organs* of *Secretion*, which separate various matters from the blood. Then follow the *Generative Organs*, which, though commonly formed into a distinct class from the Vital, ought not, by any means, to be so reckoned. For, it is evident, that they form, as it were, the sequel of the Vital Organs, being dependant on secretion, the last of the vital functions, and destined merely to propagate Vitality, and to communicate it to a new series of beings.—Under the INTELLECTUAL ORGANS, I class, first, the *Organs* of *Sense*, where impressions take place; second, the *Brain* or *Organ* of Judgement, where these excite ideas; and third, the *Nerves*, where volition results from the last. TABLE I. represents this arrangement.

This is a NATURAL ARRANGEMENT of the Anatomy of Animals, and its peculiar simplicity is illustrated by its involving, in application, that of Minerals and Vegetables, and by its being capable of instant adaption to Physiological Science.

In order to arrange Animal PHYSIOLOGY, it is only necessary to substitute the term "Functions" for "Organs;" and that science will likewise involve, in application, the Physiology of *Mineral* and *Vegetable* Bodies, and be, in its turn, capable of instant adaption to *Medical Science*.

Thus the functions are divided into *Mechanical*, *Vital*, and *Intellectual*.—The MECHANICAL FUNCTIONS are subdivided into the function of *Support*, the function of *Connection*, and the function of *Loco-motion*. The VITAL FUNCTIONS are divided into the function of *Absorption*, the function of *Circulation*, and the function of *Secretion*. The INTELLECTUAL FUNCTIONS are divided into the function of *Sensation*, the function of *Mental Operation*, and the function of *Volition*. TABLE II. exhibits this arrangement.

A circle of Functions, I may observe, thus exist in Animals, which exist not in Minerals or Vegetables; because volition, the last of the intellectual functions, connects itself to the mechanical functions, by rendering them subservient to it in Loco-motion. Thus the first and the last of these functions are as intimately connected as any of the intermediate ones, and a beautiful circle of organic function and influence is formed.

In

[ To Face Page 44. ]

### TABLE I.

### NATURAL ARRANGEMENT OF ANATOMY.

- CLASS I. Mechanical Organs.
  - ORDER I. Bones, or Supporting Organs.
  - ORDER II. Ligaments, or Connecting Organs.
  - ORDER III. Muscles, or Moving Organs.
- CLASS II. Vital Organs.
  - ORDER I. Lymphatics, &c. or Absorbing Organs.
  - ORDER II. Blood Vessels, &c. or Circulating Organs.
  - ORDER III. Glands, &c. or Secreting Organs.
- CLASS III. Intellectual Organs.
  - ORDER I. Eye, Ear, Skin, &c. or Organs of Sensation.
  - ORDER II. Brain, or Organ of Mental Operation.
  - ORDER III. Spinal Marrow, &c. or Organs of Volition.

### TABLE II.

### NATURAL ARRANGEMENT OF PHYSIOLOGY.

- CLASS I. Mechanical Functions.
  - ORDER I. Function of the Bones, or Support.
  - ORDER II. Function of the Ligaments, or Connection.
  - ORDER III. Function of the Muscles, or Motion.
- CLASS II. Vital Functions.
  - ORDER I. Function of the Lymphatics, &c. or Absorption.
  - ORDER II. Function of the Blood Vessels, &c. or Circulation.
  - ORDER III. Function of the Glands, &c. or Secretion.
- CLASS III. Intellectual Functions.
  - ORDER I. Function of the Eye, Ear, &c. or Sensation.
  - ORDER II. Function of the Brain, or Mental Operation.
  - ORDER III. Function of the Spinal Marrow, &c. or Volition.

### TABLE III.

### NATURAL ARRANGEMENT OF DISEASES.

- CLASS I. Difeafes of the Mechanical Functions.

  - ORDER I. Difeafes of Support.

    - GENUS I. Difeafes of Diminifhed Support.

    - GENUS II. Difeafes of Depraved Support.

    - GENUS III. Difeafes of Increafed Support.

  - ORDER II. Difeafes of Connection.

    - GENUS I. Difeafes of Diminifhed Connection.

    - GENUS II. Difeafes of Depraved Connection.

    - GENUS III. Difeafes of Increafed Connection.

  - ORDER III. Difeafes of Motion.

    - GENUS I. Difeafes of Diminifhed Motion.

    - GENUS II. Difeafes of Depraved Motion.

    - GENUS III. Difeafes of Increafed Motion.

- CLASS II. Difeafes of the Vital Functions.

  - ORDER I. Difeafes of Abforption.

    - GENUS I. Difeafes of Diminifhed Abforption.

    - GENUS II. Difeafes of Depraved Abforption.

    - GENUS III. Difeafes of Increafed Abforption.

  - ORDER II. Difeafes of Circulation.

    - GENUS I. Difeafes of Diminifhed Circulation.

    - GENUS II. Difeafes of Depraved Circulation.

    - GENUS III. Difeafes of Increafed Circulation.

  - ORDER III. Difeafes of Secretion.

    - GENUS I. Difeafes of Diminifhed Secretion.

    - GENUS II. Difeafes of Depraved Secretion.

    - GENUS III. Difeafes of Increafed Secretion.

- CLASS III. Difeafes of the Intellectual Functions.

  - ORDER I. Difeafes of Senfation.

    - GENUS I. Difeafes of Diminifhed Senfation.

    - GENUS II. Difeafes of Depraved Senfation.

    - GENUS III. Difeafes of Increafed Senfation.

  - ORDER II. Difeafes of Mental Operation.

    - GENUS I. Difeafes of Diminifhed Mental Operation.

    - GENUS II. Difeafes of Depraved Mental Operation.

    - GENUS III. Difeafes of Increafed Mental Operation.

  - ORDER III. Difeafes of Volition.

    - GENUS I. Difeafes of Diminifhed Volition.

    - GENUS II. Difeafes of Depraved Volition.

    - GENUS III. Difeafes of Increafed Volition.

### TABLE IV.

### NATURAL ARRANGEMENT OF THE ARTICLES OF THE MATERIA MEDICA, AS NOXIOUS POWERS.

- **CLASS I.** Medicines affecting the Mechanical Functions.
  - **Order I.** Medicines affecting the Function of Support.
    - Genus I. Medicines Diminishing Support.
    - Genus II. Medicines Depraving Support.
    - Genus III. Medicines Increasing Support.
  - **Order II.** Medicines affecting the Function of Connection.
    - Genus I. Medicines Diminishing Connection.
    - Genus II. Medicines Depraving Connection.
    - Genus III. Medicines Increasing Connection.
  - **Order III.** Medicines affecting the Function of Motion.
    - Genus I. Medicines Diminishing Motion.
    - Genus II. Medicines Depraving Motion.
    - Genus III. Medicines Increasing Motion.
- **CLASS II.** Medicines affecting the Vital Functions.
  - **Order I.** Medicines affecting the Function of Absorption.
    - Genus I. Medicines Diminishing Absorption.
    - Genus II. Medicines Depraving Absorption.
    - Genus III. Medicines Increasing Absorption.
  - **Order II.** Medicines affecting the Function of Circulation.
    - Genus I. Medicines Diminishing Circulation.
    - Genus II. Medicines Depraving Circulation.
    - Genus III. Medicines Increasing Circulation.
  - **Order III.** Medicines affecting the Function of Secretion.
    - Genus I. Medicines Diminishing Secretion.
    - Genus II. Medicines Depraving Secretion.
    - Genus III. Medicines Increasing Secretion.
- **CLASS III.** Medicines affecting the Intellectual Functions.
  - **Order I.** Medicines affecting the Function of Sensation.
    - Genus I. Medicines Diminishing Sensation.
    - Genus II. Medicines Depraving Sensation.
    - Genus III. Medicines Increasing Sensation.
  - **Order II.** Medicines affecting the Function of Mental Operation.
    - Genus I. Medicines Diminishing Mental Operation.
    - Genus II. Medicines Depraving Mental Operation.
    - Genus III. Medicines Increasing Mental Operation.
  - **Order III.** Medicines affecting the Function of Volition.
    - Genus I. Medicines Diminishing Volition.
    - Genus II. Medicines Depraving Volition.
    - Genus III. Medicines Increasing Volition.

### TABLE V.

### NATURAL ARRANGEMENT OF THE ARTICLES OF THE MATERIA MEDICA, AS REMEDIES.

- **CLASS I.** Medicines affecting the Mechanical Functions.

  - **Order I.** Medicines affecting Support.

    - Genus I. Medicines Increasing Support.

    - Genus II. Medicines Rectifying Support.

    - Genus III. Medicines Diminishing Support.

  - **Order II.** Medicines affecting Connection.

    - Genus I. Medicines Increasing Connection.

    - Genus II. Medicines Rectifying Connection.

    - Genus III. Medicines Diminishing Connection.

  - **Order III.** Medicines affecting Motion.

    - Genus I. Medicines Increasing Motion.

    - Genus II. Medicines Rectifying Motion.

    - Genus III. Medicines Diminishing Motion.

- **CLASS II.** Medicines affecting the Vital Functions.

  - **Order I.** Medicines affecting Absorption.

    - Genus I. Medicines Increasing Absorption.

    - Genus II. Medicines Rectifying Absorption.

    - Genus III. Medicines Diminishing Absorption.

  - **Order II.** Medicines affecting Circulation.

    - Genus I. Medicines Increasing Circulation.

    - Genus II. Medicines Rectifying Circulation.

    - Genus III. Medicines Diminishing Circulation.

  - **Order III.** Medicines affecting Secretion.

    - Genus I. Medicines Increasing Secretion.

    - Genus II. Medicines Rectifying Secretion.

    - Genus II. Medicines Diminishing Secretion.

- **CLASS III.** Medicines affecting the Intellectual Functions.

  - **Order I.** Medicines affecting Sensation.

    - Genus I. Medicines Increasing Sensation.

    - Genus II. Medicines Rectifying Sensation.

    - Genus III. Medicines Diminishing Sensation.

  - **Order II.** Medicines affecting Mental Operation.

    - Genus I. Medicines Increasing Mental Operation.

    - Genus II. Medicines Rectifying Mental Operation.

    - Genus III. Medicines Diminishing Mental Operation.

  - **Order III.** Medicines affecting Volition.

    - Genus I. Medicines Increasing Volition.

    - Genus II. Medicines Rectifying Volition.

    - Genus III. Medicines Diminishing Volition.

In order to arrange MEDICAL SCIENCE, for the term "HEALTHY FUNCTIONS," the subject of physiology, it is only necessary to substitute the term "Diseased Functions." The Classes of Disease are, therefore, like those of Anatomy and Physiology, three; namely, DISEASES OF THE MECHANICAL OR LOCO-MOTIVE FUNCTIONS, DISEASES OF THE VITAL FUNCTIONS, AND DISEASES OF THE INTELLECTUAL FUNCTION. The ORDERS of the first Class, as affecting the functions of the *Bones*, the *Ligaments*, and the *Muscles*, are three, viz. DISEASES OF SUPPORT, DISEASES OF CONNECTION, AND DISEASES OF LOCO-MOTION. Those of the second Class, as affecting the functions of the *Absorbent, Circulating*, and *Secreting* Vessels, are likewise three, viz. DISEASES OF ABSORPTION, DISEASES OF CIRCULATION, and DISEASES OF SECRETION. Those of the third Class, as affecting the functions of the *Organs of Sense*, of the *Brain*, and of the *Nerves*, are also three, viz. DISEASES OF IMPRESSION, DISEASES OF JUDGEMENT, and DISEASES OF VOLITION. The *Genera*, under each order, consist of *Diseases* of *Diminished, Irregular*, and *Increased Function*, as will readily be understood from TABLE III.

This arrangement of disease likewise involves in application, that of Minerals (if we choose to maintain the analogy, and to give that name to mere injury of structure) and of Vegetables, as well as Animals. The first would have what might be termed Diseases of Mechanical Structure; the second, Diseases of Mechanical and Vital Functions; and the third, Diseases of Mechanical, Vital, and Intellectual Functions; corresponding to the observation, that Minerals exist, vegetables exist and live, and animals exist, live, and think.

Precisely in the same way would I class the articles of the Materia Medica; first, as operating upon the Mechanical, Vital, or Intellectual Organs, and then as either diminishing, depraving, or increasing their action.—TABLE IV. exhibits an arrangement of them as Noxious Powers; because, in it, their genera *correspond* to the genera of disease. TABLE V. exhibits an arrangement of them as Remedies, because, in it, their genera are *contrasted* or *opposed* to the genera of disease. In the first the genera are arranged under the heads "Diminishing, Depraving*, Increasing;" in the second under the heads, "Increasing, Rectifying*, Diminishing."

By

* It will be obvious, that it is entirely from the want of appropriate terms, that these words are used with considerable latitude.

By comparing them with the table of Diseases, their use will be obvious.

### DIVISION V.

### *Remarks upon the Natural Arrangement.*

The correctness of this arrangement, as strictly founded on physiology, is confirmed by the following observations.

Disease is change of healthy state and healthy action. It is change of state, as well as action, because action depends on state, life itself on organization; and it is only by affecting structure, the objects acting, that change of action can be produced. In fracture, as far as the bone is concerned, the action peculiar to it, Loco-motion, is affected; as the blood vessels are ruptured, their action is affected; as the nerves are injured, their peculiar action is also affected. Thus each has its peculiar actions, and the action of each is affected by means of its structure; but the affection of the state and action of the vessels or nerves, is not, in this case, the primary disease, but only an extension of it to other parts or other functions; or, more properly, they are new diseases, because they affect new functions, and are, in their nature, entirely different.

Now, if disease consists in change of function, different diseases must either consist in different changes of the same function, or in the change of different functions; the former, as being a more minute division, making evidently the genera; the latter, as being more general the orders.

From the inspection of the Table, and a consideration of the nature of disease, it will appear, that whatever is commonly reckoned one disease, is, generally, perhaps always, three; thus fracture is always accompanied by inflammation and pain; and, while the simple fracture is a disease of diminished support, the inflammation accompanying it is a disease of increased circulation; and the pain accompanying it a disease of increased impression.

With regard to the articles of the materia medica, the correctness of my arrangement, as indicating their influence upon the different classes of organs, will, on the slightest consideration, appear equally evident. Caustic, for instance, affects the mechanical, mercury the vital, and opium the intellectual system. But, like the diseases which they remedy, it will appear, that each of these also affects all of the functions, though in a less direct manner than that which I have just now particularly pointed out as its object, and upon which the exact place of each, in a systematic arrangement, ought to depend.

It cannot, therefore, be a just objection to this arrangement that it throws each of what are commonly called diseases under three different classes, as, in reality, each of the common diseases is more properly three, and exists under three natural classes. Even in this way does every physician, though unaware of it, to a certain, though limited extent, consider them; and, therefore, instead of giving any new complexity to nosology, it merely explains and simplifies that complexity which ever has existed in the nature of disease. In other words, it is natural, and developes only the combinations of nature.

Thus no one disease can, with the slightest truth, be said to fall under three different classes, as inflammatory and suppurative phthisis, which are even commonly reckoned distinct stages, or as fracture, inflammation, &c. which are essentially different in their nature, and may be combined in every possible form; thus it can serve no useful purpose to reckon them one, while it destroys every natural arrangement, and all the invaluable consequences dependant on it.

Although, however, nosology must in any thing like a natural arrangement be thus fixed most accurately upon the basis of physiology, the classification of every disease may be derived from the particular derangement of that individual function which occasions the derangement of all the rest, and not from the derangement of all the three functious, which would probably render names uselessly complex.—I say uselessly, because as the whole of any one disease is primarily dependant on the derangement of one function, so health is to be recovered chiefly by the restoration of that function, and the mode of cure is chiefly indicated by the name of the genus under which the disease is primarily arranged. This last is one of the most important consequences of such a system.

If, however, the names of diseases and their classification are not thus made dependant most strictly on *each specific change from* the healthy state described in physiology, they are at once arbitrary and unnatural; the pathology and nosology, of which they constitute a part, has not its foundation in physiology; nor is the practice constructed upon them indicated by the reasonings they deliver.

To reckon a series of occurrences which entirely differ in in their nature, consequences, and mode of cure, as one disease, may serve the purposes of common language, but by no means those of medical science, or of medical arrangement.

In considering the Natural Arrangement of Medical Science exhibited above, it will be particulary necessary to avoid substituting the term "organ" for "function." Diseases of organs express merely the seats of disease; diseases of functions express their nature. Nevertheless, a reference may also be made to the various organs, as it is evident that there may be disease of the vital, or any other function in a mechanical organ; thus a tumour in a muscle is a disease of Secretion, and an ulcer in a similar situation a diease of Absorption; both being orders of the class, Diseases of the Vital Functions.

### Division VI.

### *Results from the Adoption of the System Proposed, &c.*

The most valuable result of the adoption of the Natural System is, that it would be possible for the physician to refer to the several columns of a table, each of which, with regard to the dieases which it enumerates, is precisely similar in its nature; indicates, correspondingly arranged, articles of the Materia Medica, and requires precisely a similar method of cure.—From it consequently flows the simplest

### First Principle of the Medical Art.

This Principle is founded upon a knowlege of the relations subsisting between the various states of the Animal Functions, and of the Powers of Medicines, *as exhibited in the preceding tables;* and it precisely consists in the application of the articles of the different genera of Medicines* to the cure of the *opposite* genera of Disease†.

In this Natural System it will be observed, that all its parts correctly correspond, and beautifully flow from each other; while Anatomy is the basis of Physiology, Physiology, in its turn, is the basis of Medicine; the classification of one is applicable to the rest, and all of them at once involve Mineral, Vegetable, and Animal nature.—This system will, I trust, ultimately shed an useful light over anatomical, over physiological, and particularly overthe dark paths of medical science. But it must be remembered,

---

* As arranged in Table V. † As arranged in Table III.

ed that this paper exhibits merely a sketch of it, and that sketch necessarily brief, and deprived of every illustration. Yet, I think, I may conclude it by adding the words of Lord Bacon, that "the harmony of a science, supporting each part the other, is, and ought to be, the true and brief confutation and suppression of all the smaller sort of objections.‡"

‡ Advancement of Learning, Book I.

